# Genotyping of rabbit hemorrhagic disease virus detected in diseased rabbits in Egyptian Provinces by *VP60* sequencing

**DOI:** 10.14202/vetworld.2020.1098-1107

**Published:** 2020-06-15

**Authors:** Ahmed M. Erfan, Azhar G. Shalaby

**Affiliations:** Reference Laboratory for Veterinary Quality Control on Poultry Production, Animal Health Research Institute, Dokki. Agricultural Research Centre, Giza, 12618, Egypt

**Keywords:** Egypt, native rabbits, prevalence, rabbit hemorrhagic disease virus, *VP60*

## Abstract

**Background and Aim:**

Rabbit hemorrhagic disease (RHD) is an economically important disorder of rabbits, where infection results in severe losses to the meat and fur industries. Our goal was to characterize the RHD virus (RHDV) strains currently circulating in different regions of Egypt.

**Materials and Methods:**

Fifty rabbits suspected of harboring RHDV from 15 Egyptian governorates were evaluated. Diseased rabbits were identified by clinical signs and postmortem lesions. RHDV was confirmed through hemagglutination assay (HA) and polymerase chain reaction (PCR). Partial sequencing of the *VP*60 gene was performed for genotyping.

**Results:**

From 50 rabbits, we identified 16 cases of RHDV (32%) by HA and PCR, including seven males and nine females. We identified two distinct genotypes through sequencing of an amplified fragment of the virus *VP60* gene. One group is composed of those circulating primarily in upper Egypt, which is closely related to the classical G3-G5 virus strains, and the second group, circulating predominantly in lower Egypt, was more closely related to the RHDV2 variant. The overall nucleotide sequence identity ranged from 78.4% to 100%, and identity with the vaccine strains ranged from 78.8% to 91.1%.

**Conclusion:**

Our results constitute important documentation of RHDV strains currently circulating in Egypt. The findings suggest that there may be a limit to the effectiveness of currently applied vaccine strains as this formulation may not cover all circulating strains. A wider investigation that includes both domestic and wild rabbits will be needed to identify appropriate control measures for this disease.

## Introduction

The first case of rabbit hemorrhagic disease virus (RHDV) in Egypt was recorded in Sharkia Province in 1991, and this disease has since spread to other Egyptian governorates [1]. Despite active vaccination programs, RHDV remains a threat to farms that produce rabbit meat for popular consumption [2] because of high disease-associated morbidity and mortality.

Rabbit hemorrhagic disease (RHD) is a rapidly fatal viral disease of adult rabbits. The first recorded clinical cases of RHD were in China [3]. The disease spread rapidly through Asia and Europe and became endemic within just few years [4-6]. The etiological agent, RHDV, is a member of the Calicivirus family. Typically, RHDV kills more than 90% of susceptible adult animals within 2-3 days of infection [6]. In 2010, a new strain, RHDV2, was identified in association with infection of rabbits of different ages and populations in France [7]. RHDV2 spread rapidly throughout Europe [8,9] and was detected in Australia in 2015 [10] and in Canada in 2016 [11]. RHDV2 is now considered endemic in Europe [12]. The disease associated with RHDV2 includes symptoms that are similar to those of classic RHDV, although mortality rates are comparatively low [13]. Transmission of RHDV can be through oral, nasal, and conjunctival routes, and transmission through a vector-like insect has been reported [14]. Transmission of RHDV may also occur through direct contact with an infected animal as infected rabbits may shed the virus in their excretions [15]. The pathogenesis of RHD includes development of petechial hemorrhages in multiple systemic organs as a result of virus-induced hyper-coagulopathy, and the most severe form of these lesions appear in the liver, trachea, and lungs [16]. RHDV also promotes fatal hepatitis, most notably in adult rabbits [7]. RHDV2 can result in fatal infections of rabbits at different ages, including those as young as 11 days old [17]. This new lagovirus has also been detected in wild hares [18,19]. The combination of classical and variant viruses by definition has resulted in increased diversity in this virus species [20,21]. The genomic structures of RHDV and RHDV2 are similarly arranged, and both include two open reading frames (ORFs). ORF1 encodes the nonstructural proteins, including the RNA-dependent RNA ­polymerase and the major capsid protein (VP60). The second ORF encodes a minor structural protein known as *vp10* [22,23]. Many polymerase chain reaction (PCR)-based methods are available for the identification of viruses, including rabbit viruses, and these methods are substantially more rapid than traditional virus isolation as they offer the possibility of high throughput with improved sensitivity and specificity. PCR also facilitates virus gene sequencing so that all vaccine and wild-type virus strains can be fully typed and differentiated. PCR can also be used for the detection of pathogens that are difficult to be isolated using traditional methods and those that are present in low titer in test samples [24]. The virus *VP60* gene has been selected as a target [25] for reverse-transcription PCR (RT-PCR) assay which is currently used as a general screening tool for rabbit and hare caliciviruses. The *VP60* gene was also used for the development of a universal RT-PCR method for detecting lagoviruses that uses primers that span a highly conserved region [26].

The aim of the current study was to identify circulating RHDV strains found in distinct populations of rabbits in Egypt. Our goal is to use molecular analyses to determine the relationship of any new isolates to the strains currently used for the critical vaccination strategies.

## Materials and Methods

### Ethical approval

This study does not contain any experimental studies with animals. Diseased rabbits were euthanized before sampling in accordance with the regulations of the General Organization for Veterinary Services and Animal Health Research Institute.

### Study period and location

The study was performed during 2018-2019 along 15 representative Governorates.

### Sample collection

Lung and liver samples were collected from 10 rabbits per flock after intravenous administration of 3-5 mg/kg xylazine and 35-40 mg/kg ketamine to facilitate euthanasia. All tissue samples were collected from all freshly killed rabbits. The surveyed populations were of various ages and sexes. All samples were collected aseptically, placed in sterile bags, and transported to the lab on dry ice for further laboratory evaluation. The samples were collected from rabbits in 15 different Egyptian governorates, as listed in Table-1.

**Table-1:** The sources of examined samples for RHDV.

No.	Egyptian governorates	Total no of rabbits in flocks	No of examined suspected flock	Source of samples			

				Male	Vaccination status	Female	Vaccination status
1.	Cairo	100	4	1	unvacc	3	unvacc
2.	Giza	209	6	1	unvacc	5	unvacc
3.	Fayom	74	4	1	unvacc	3	unvacc
4.	Kaliobia	36	2	1	unvacc	1	unvacc
5.	Behira	205	3	0	0	3	2 vacc
6.	Sharkia	385	5	2	unvacc	3	3 vacc
7.	Gharbia	290	7	2	unvacc	5	1 vacc
8.	Menofia	23	2	1	unvacc	1	unvacc
9.	khafr el Sheikh	60	2	1	unvacc	1	unvacc
10.	Alex	40	1	0	0	1	unvacc
11.	Dakhlia	107	4	0	0	4	unvvacc
12.	Menia	96	5	3	unvacc	2	unvacc
13.	Sohag	60	1	0	0	1	unvacc
14.	Assuit	71	3	1	unvacc	2	unvacc
15.	Qina	20	1	0	0	1	unvacc
Total		1776	50	14	-	36	6 vacc

RHDV=Rabbit hemorrhagic disease virus

### Hemagglutination assay (HA)

An HA was performed according to previously published methods [27]. Briefly, a fragment of liver tissue was mechanically homogenized in 10-20% (w/v) saline solution (pH 7.2), followed by clarification of the supernatant by centrifugation at 5000 g for 10 min. Type O human red blood cells (RBCs; VACSERA, Cairo, Egypt) were washed in phosphate-buffered saline (PBS; pH 6.5) and then subjected to centrifugation at 500 g for 10 min. The sedimented erythrocytes were re-suspended at 0.75% concentration in PBS (pH 7.2). HA was performed with serial two-fold dilutions of clarified liver homogenate in 50 ml PBS, pH 7.2, and positive and negative controls were also included. Fifty microliters of 0.75% washed type O human RBCs were added to each well, followed by incubation at 4°C for 1 h. The HA titer was determined according to the reciprocal of the highest dilution capable of generating hemagglutination of the RBCs.

### RNA extraction

RNA extraction from the clarified tissue homogenates was performed using the QIAamp viral RNA Mini kit (Qiagen, Gmbh, Germany) according to the manufacturer’s instructions. Briefly, 140 µl of the supernatant was incubated with 560 µl of AVL lysis buffer and 5.6 µl of carrier RNA at room temperature for 10 min. After incubation, 560 µl of 100% ethanol was added. The sample was then washed and centrifuged 2 times. RNA was eluted with 60 µl of elution buffer.

### PCR amplification

Oligonucleotide primers (Metabion, Germany) that were designed [28] to amplify 538 bp of the *VP60* gene (P33: CCACCACCAACACTTCAGGT and P34: CAGGTTGAACACGAGTGTGC) were used in a 25 µl reaction containing 12.5 µl of Quantitect probe RT-PCR buffer (Qiagen, Gmbh, Germany), 1 µl of each of the primers at a concentration of 20 pmol, 0.25 µl of reverse transcriptase, 7.25 µl of water, and 3 µl of RNA template. The reaction was performed in a Biometra thermal cycler. Reverse transcription was carried out at 50°C for 30 min, followed by a primary denaturation step at 95°C for 15 min, 35 cycles of 94°C for 30 s, 56°C for 40 s, and 72°C for 45 s. A final extension step was performed at 72°C for 10 min.

### Analysis of the PCR products

Fifteen microliters of the amplified *VP60* PCR products were evaluated by gel electrophoresis using ultrapure 1.5% agarose (Invitrogen, Thermo Fisher Scientific, Germany) in 1×Tris-borate-EDTA (TBE) buffer at room temperature. Gelpilot 100 bp DNA ladder (Qiagen, Gmbh, Germany) was used to determine fragment sizes. PCR-amplified bands were detected by imaging using a gel documentation system (Alpha Innotech, Biometra). Data were analyzed using Automatic Image Capture Software (Protein Simple, formerly Cell Biosciences, San Jose, CA, USA).

### Gene sequencing

#### Genetic and phylogenetic analyses

PCR products were purified using a QIAquick PCR Product extraction kit (Qiagen, Gmbh, Germany). Sequence reactions were performed using a Bigdye Terminator V3.1 cycle sequencing kit (Perkin-Elmer), and purification was performed using Centri-Sep spin columns (Thermo Fisher, Germany). VP60 sequences were obtained using a 3130 genetic analyzer (Applied Bio-systems, Life technologies, Thermo Fisher, Germany). Basic Local Alignment Search Tool (BLAST^®^) [29] alignment was performed to establish sequence similarities to the sequences deposited in the GenBank database. The MegAlign module of Lasergene DNA-Star ver­sion 12.1 was used to determine phylogenetic dis­tances among the analyzed strains [30], and MEGA6 was used to create a phylogenetic tree using max­imum composite likelihood with 1000 bootstrap replications, neighbor-joining, and maximum parsi­mony [31].

## Results

**Figure-1:**
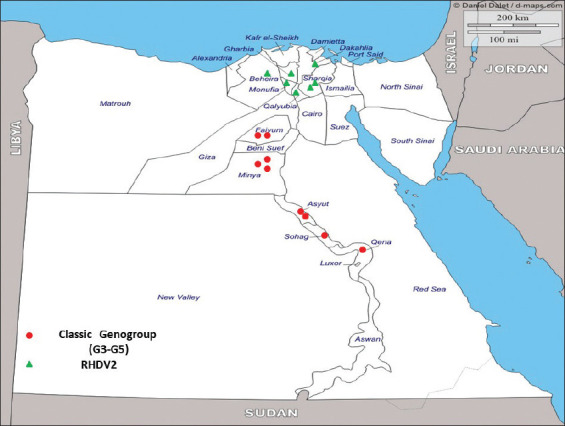
Egyptian epidemiological map for rabbit hemorrhagic disease virus (RHDV) according to the geographical distribution during (2018-2019). The classical form of RHDV virus appeared as red dots, while RHDV2 appeared green triangle and mainly distributed in the lower Egypt [Source: https://www.d-maps.com].

In this study, the diagnosis of suspected RHDV cases was determined based on disease-associated clinical signs, postmortem lesions, HA activity, conventional PCR, and sequencing of the RHDV *VP60* gene. A total of 50 rabbit herds were initially identified as suspected RHDV, and this suspicion was based on records of sudden death in rabbit flocks associated with neurologic and respiratory signs together with liver necrosis and generalized petechial hemorrhages in multiple tissues as revealed by postmortem examination. These animals surveyed from 15 different governorates in Egypt. Only six flocks of female rabbits had been vaccinated against RHDV, and all 16 rabbits ultimately diagnosed for RHDV were from flocks that were unvaccinated (Table-1, Supplementary Tables-S1 and S2 and Figure-1).

**Supplementary Table-1:** Collection data for positive samples.

Sample	Age	Governorate	Sample	Collection date	Sex	Quantitative plate HA result	HA titer	Vaccination status	No. of rabbits
1	3 weeks	Kaliobia	Lung and Liver	March 2018	Male	+	2^12^	Unvacc	20
2	12 weeks	Faium	Liver	July 2019	Female	+	2^10^	Unvacc	12
3	8 weeks	Menia	Lung and Liver	July 2019	Male	+	2^14^	Unvacc	30
4	6 weeks	Sharkia	Liver	June 2018	Male	+	2^11^	Unvacc	35
5	4 weeks	Sharkia	Liver	June 2018	Male	+	2^13^	Unvacc	50
6	7 weeks	Menia	Liver	July 2019	Male	+	2^12^	Unvacc	18
7	6 weeks	Assiut	Lung and Liver	July 2019	Female	+	2^16^	Unvacc	20
8	2 weeks	Menofia	Lung and Liver	July 2019	Male	+	2^12^	Unvacc	5
9	8 weeks	Gharbia	Liver	February 2019	Female	+	2^9^	Unvacc	10
10	10 weeks	Qena	Lung and Liver	April 2019	Female	+	2^14^	Unvacc	20
11	18 days	Dakahlia	Lung and Liver	April 2019	Female	+	2^11^	Unvacc	30
12	9 weeks	Assiut	Lung and Liver	January 2019	Male	+	2^11^	Unvacc	40
13	10 weeks	Sohag	Liver	August 2018	Female	+	2^13^	Unvacc	60
14	12 weeks	Behira	Liver	February 2019	Female	+	2^14^	Unvacc	25
15	6 weeks	Faium	Liver	December 2018	Female	+	2^10^	Unvacc	20
16	8 weeks	Menia	Liver	August 2018	Female	+	2^12^	Unvacc	9

HA=Hemagglutination assay

**Supplementary Table-2:** Collection data for negative samples.

Sample	Age	Governorate	Sample	Collection date	Sex	Vaccination status	No. of rabbits
1	4 weeks	Cairo	Lung and Liver	May 2019	Male	Unvacc	50
2	10 weeks	Cairo	Lung and Liver	May 2019	Female	Unvacc	30
3	3 weeks	Cairo	Lung and Liver	May 2019	Female	Unvacc	10
4	1 month	Cairo	Lung and Liver	May 2019	Female	Unvacc	10
5	3 weeks	Giza	Lung and Liver	December 2018	male	Unvacc	15
6	19 days	Giza	Lung and Liver	November 2018	Female	Giza-2006	100
7	2 months	Assiut	Liver	July 2019	Female	Unvacc	11
8	1 month	Behira	Lung and Liver	February 2019	Female	Giza-2006	120
9	3 months	Kaliobia	Lung and Liver	March 2018	Female	Unvacc	16
10	2 months	Menia	Lung and Liver	August 2018	Female	Unvacc	27
11	1 month	Menia	Lung and Liver	August 2018	Male	Unvacc	12
12	7 weeks	Gharbia	Liver	February 2019	Male	Unvacc	40
13	3 months	Gharbia	Liver	February 2019	Female	Giza-2006	150
14	1 month	Giza	Lung and Liver	May 2019	Female	Unvacc	40
15	5 weeks	Giza	Lung and Liver	May 2019	Female	Unvacc	30
16	2 weeks	Giza	Lung and Liver	May 2019	Female	Unvacc	9
17	3 months	Giza	Lung and Liver	May 2019	Female	Unvacc	15
18	2 months	Kafrelsheikh	Lung and Liver	January 2019	male	Unvacc	20
19	5 weeks	Behira	Lung and Liver	April 2019	Female	Giza-2006	60
20	6 weeks	Kafrelsheikh	Lung and Liver	March 2019	Female	Unvacc	50
21	9 weeks	Alex	Lung and Liver	June 2019	Female	Unvacc	40
22	2 weeks	Menofia	Lung and Liver	February 2019	Female	Unvacc	18
23	17 days	Gharbia	Liver	January 2019	Female	Unvacc	22
24	3 months	Gharbia	Liver	January 2019	male	Unvacc	31
25	1 month	Gharbia	Liver	January 2019	Female	Unvacc	20
26	6 weeks	Gharbia	Liver	January 2019	Female	Unvacc	7
27	7 weeks	Dakahlia	Lung and Liver	October 2018	Female	Unvacc	27
28	2 months	Dakahlia	Lung and Liver	October 2018	Female	Unvacc	30
29	1 month	Dakahlia	Lung and Liver	October 2018	Female	Unvacc	20
30	5 weeks	Sharkia	Lung and Liver	June 2018	Female	Giza-2006	90
31	3 months	Sharkia	Lung and Liver	June 2018	Female	Giza-2006	70
32	20 days	Sharkia	Lung and Liver	June 2018	Female	Giza-2006	200
33	9 weeks	Faium	Lung and Liver	December 2018	Female	Unvacc	18
34	5 weeks	Faium	Lung and Liver	December 2018	Male	Unvacc	24

As shown in Figure-1, the flocks that were RHDV-positive were from the upper Egyptian governorates, and those diagnosed with RHDV2 were from the lower Egyptian provinces.

### VP60 PCR

A specific 538 bp band encoding a fragment of the virus *VP60* gene was amplified by PCR in 16 of the 50 rabbit herds tested (32%). Fifty percent of the males tested were found to be RHDV-positive, in contrast to only 25% of females tested (Table-2).

**Table-2:** Incidence of RHDV in both examined sexes.

Sum of no. of cases	Cases			

	Negative	Positive	Total	Positive RHDV percentage
Female	27	9	36	25
Male	7	7	14	50
Grand total	34	16	50	32

RHDV=Rabbit hemorrhagic disease virus

### HA activity

All of the 16 samples from RHDV-PCR-positive rabbits also tested positive for HA activity. HA titers varied from 2^9^ to 2^16^.

### DNA sequencing

**Figure-2:**
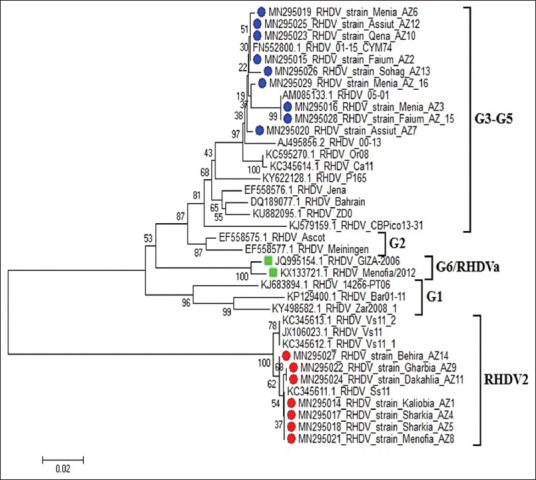
The phylogenetic tree of sequenced strains (AZ1 and AZ16) and other randomly selected strains form GenBank (MEGA 6 – neighbor-joining and maximum parsimony tool) [31].

DNA sequencing of the *VP60* amplicons revealed a range of 78.4-100% nucleotide sequence identity. Sequence analysis indicated that two distinct RHDV types were circulating in different districts in Egypt. A sequence from one set of amplicons was closely related to that of the classical G3-G5 strains, and the other set of amplicons was more closely related to RHDV2 (Figure-2). The new sequences were assigned GenBank accession numbers MN295014-MN295029. In 2018, both RHDV genotypes (RHDV group G3-G5 and RHDV2) were detected equally. In 2019, RHDV2 was identified more frequently, as shown in Table-3. The overall nucleotide sequence identity with the Egyptian RHDV-vaccine strains (GenBank accessions numbers KX133721.1 and JQ995154.1) ranged from 78.8% to 91.1%.

**Table-3:** A collective data for the positive cases with reference to the year of detection, governorates and strain type.

Flock no	Age	Age group	Governorate	Sex	Collection date	Genbank acc. no. and code	Genogroup	Quantitative plate HA result	HA titer
1	3 weeks	<2 months	Kaliobia	Male	March 2018	MN295014 AZ1	RHDV2	+	2^12^
2	12 weeks	2 months and more	Faium	Female	July 2019	MN295015 AZ2	Classic RHDV Genogroup5	+	2^10^
3	8 weeks	2 months and more	Menia	Male	July 2019	MN295016 AZ3	Classic RHDV Genogroup5	+	2^14^
4	6 weeks	<2 months	Sharkia	Male	June 2018	MN295017 AZ4	RHDV2	+	2^11^
5	4 weeks	<2 months	Sharkia	Male	June 2018	MN295018 AZ5	RHDV2	+	2^13^
6	7 weeks	<2 months	Menia	Male	July 2019	MN295019 AZ6	Classic RHDV Genogroup5	+	2^12^
7	6 weeks	<2 months	Assiut	Female	July 2019	MN295020 AZ7	Classic RHDV Genogroup5	+	2^16^
8	2 weeks	<2 months	Menofia	Male	July 2019	MN295021 AZ8	RHDV2	+	2^12^
9	8 weeks	2 months and more	Gharbia	Female	February 2019	MN295022 AZ9	RHDV2	+	2^9^
10	10 weeks	2 months and more	Qena	Female	April 2019	MN295023 AZ10	Classic RHDV Genogroup5	+	2^14^
11	18 days	<2 months	Dakahlia	Female	April 2019	MN295024 AZ11	RHDV2	+	2^11^
12	9 weeks	2 months and more	Assiut	Male	January 2019	MN295025 AZ12	Classic RHDV Genogroup5	+	2^11^
13	10 weeks	2 months and more	Sohag	Female	August 2018	MN295026 AZ13	Classic RHDV Genogroup5	+	2^13^
14	12 weeks	2 months and more	Behira	Female	February 2019	MN295027 AZ14	RHDV2	+	2^14^
15	6 weeks	<2 months	Faium	Female	December 2018	MN295028 AZ15	Classic RHDV Genogroup5	+	2^10^
16	8 weeks	2 months and more	Menia	Female	August 2018	MN295029 AZ16	Classic RHDV Genogroup5	+	2^12^

RHDV=Rabbit hemorrhagic disease virus, HA=Hemagglutination assay, PCR=Polymerase chain reaction

Most cases of RHDV were detected during the summer months. In 2018, infections were detected at the highest frequency (50%) during the month of August. By contrast, in 2019, 83% of RHDV infections were detected in July.

The phylogenetic tree is notable for clear clustering of the two distinct virus genotypes, and these groups can be distinguished from the two aforementioned vaccine strains, as shown in Figure-2.

### Epidemiological findings

The sequence data revealed that there are presently two genotypes of RHDV circulating in distinct regions and governorates within Egypt. As shown in Figure-1, the geographical distribution of the ­circulating strains of the RHDV virus showed that genotypes associated with the classical RHDV strain (G3-G5) were concentrated mainly in upper Egypt, and those associated with RHDV2 circulated in lower Egypt.

The RHDV-positive samples were divided into two groups according to the age of the rabbit host (<2 months vs. ≥2 months of age). As shown in Table-4, classical RHDV genotype samples represented 75% of the older group, while RHDV2 samples represented 62.5% of the younger group.

**Table-4:** The difference between age groups in relation to the detected RHDV strains.

Age groups	Classic RHDV Genogroup (5)	RHDV2 (variant)	Total positive	% of classical	% of variant RHDV-2
2 months and more (8 weeks or more)	6	2	8	6/8=75	2/8=25
<2 months (<8 weeks)	3	5	8	3/8=37.5	5/8=62.5
Total	9	7	16	9/16=56.3	7/16=43.7

RHDV=Rabbit hemorrhagic disease virus, HA=Hemagglutination assay, PCR=Polymerase chain reaction

## Discussion

According to an 2017 report from the Food and Agriculture Organization of the United Nations, Egypt is one of the five major producers of rabbit meat, where Italy was the fifth largest producer of rabbit meat in the world, following China, the Democratic People’s Republic of Korea, Spain, and Egypt [32]. In the early 1990s, RHDV was classified as a member of the family Caliciviridae [33,34], genus *Lagovirus* [6]. The virus is typically highly contagious among rabbits of different species and ages [35,36], and infection with the virus can result in sudden death that is typically associated with liver necrosis and acute hepatitis [37,38] with generalized congestion and hemorrhage [39,40]. In this study, we identified 16 cases that were positive for RHDV using PCR, and these 16 cases were hemagglutinin-positive, with titers ranging from 2^9^ to 2^16^. These results are consistent with those reported previously [13] that indicated that RHDV2 efficiently agglutinates human type O RBCs and confirmed the use of HA as a routine diagnostic tool for the detection of RHDV2 in infected samples. Conventional RT-PCR was used to detect RNA of RHDV from liver and lung samples, and 16 out of 50 cases (32%) of suspected RHDV were revealed as positive by this method. The sampled cases were from 15 Egyptian governorates and were collected in 2018-2019. The classical RHDV strain was identified primarily in localities within upper Egypt, and RHDV2 was detected primarily within the governorates of the lower Egypt. Overall, the detection of the circulating strains of both RHDV and RHDV-2 is consistent with findings reported previously [12] regarding RHDV2 as the dominant circulating strain in both wild and domestic rabbits. Interestingly, the disease associated with the RHDV2 variant manifests in a similar fashion to that caused by classical RHDV, although the mortality rates may be more variable and tend to be lower [13].

**Figure-3:**
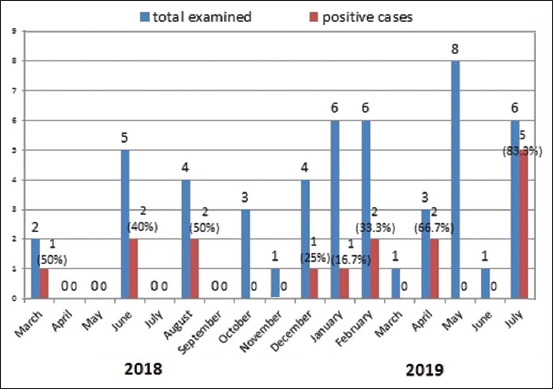
The distribution of positive cases in proportion to the total examined with relation to the month and year of detection (2018-2019) in Egypt.

An initial analysis of genetic diversity among these strains was initiated by comparing partial sequences from a few RDHVs isolated from European countries [41,42]. Our results are consistent with those reported in a previous study [43], in which rabbits <28 days old were found to be susceptible to RHDV disease. In this study, the samples were collected from rabbits at different ages and during different seasons. The group that tested positive was divided into those <2 months of age and those ≥2 months of age, as shown in Table-3. Classical RHDV was detected in three out of eight cases of the younger age group, while five cases tested positive for RHDV2. However, in the older age group, six of the eight rabbits (75%) tested pos­itive for classical RHDV. An earlier publication, [44] discussed the fact that many clinical cases have been recorded from different governorates within Egypt, with clinical signs consistent with acute RHD, and these results suggested that the two strains might have comparatively equal virulence during natural RHDV outbreaks. Regarding the seasonality of this disease, most of the cases in our study were detected during the summer season, most notably in the month of July (Figure-3). By contrast, other reports [45] noted that RHDV was identified in rabbits during the spring of 1991 in Sharkia Province associated with 90% losses. RHDV was also reported during the winter and spring of 1993 in upper Egypt (Minya, Assiut, and Sohag Provinces), with mortality rates from 26.7% to 100% among 14-16 week-old rabbits. Amplification, sequencing, and phylogenies of the *VP60* gene from all incidents, in addition to the immunological typing of the virus material from three cases, confirmed the presence of RHDV2 [43].

In this study, we amplified a 538 bp fragment of the *VP60* gene, and this has been reported to be highly conserved among RHDV variants. Our findings are consistent with those reported earlier [25] regarding the efficacy of *VP60* as a target for PCR assays and as a general screening tool for rabbit and hare caliciviruses. The *VP60* gene was used to develop universal *Lagovirus* PCR primers that span a highly conserved region within RHDV [26]. Results from another study [46] revealed that the viral *VP60* gene was targeted to explore all rabbit lagoviruses circulating in Australia. Here, we used these primers exclusively for the primary screening of animals for suspected RHDV infections. Among our findings, the *VP60* sequences from our study were most closely related to those from 2010 to 2012 RHDV2 isolates from France, Spain, and Portugal with 97%-98% nucleotide sequence identity [47]. Interestingly, RT-PCR primers targeting the *VP60* gene sequence were also used for the detection of several distinct species of lagoviruses, including the European brown hare syndrome virus and rabbit calicivirus [26].

The study analyzed the phylogeny of 16 RHDV strains detected in 2018 and 2019 from 15 different provinces in Egypt, and amplification and sequencing of a fragment of the *VP60* capsid gene from virus cDNA resulted in the identification of novel virus strains with nucleotide identities ranging from 78.4% to 100%. Among our main conclusions, we found that the two major RHDV strains, namely, RHDV and RHDV2, were circulating in different regions of Egypt. One of these strains was the classical G3-G5 RHDV, and the other strain was variant-type RHDV2 (GenBank accessions numbers MN295014-MN295029).

The rabbit industry is of importance in the production of both meat and fur, and as such, it needs to focus on ways to prevent viral, bacterial, and parasitic diseases [22,48,49]. Our results confirm infections with different strains of RHDV that is circulating concurrently within Egypt, and these viruses infect both male and female rabbits within various age groups. Our results also suggest that the current vaccine strain may not be sufficient in protecting against all strains currently in circulation. As such, research into future vaccination strategies is stressed here.

In the current study, RT-PCR amplification of a fragment of the *VP60* gene confirmed the presence of RHDV RNA in liver and lung samples of 16 cases out of 50 total suspected cases (32%), suggesting a high incidence of this disease. These findings are similar to those reported in earlier Egyptian studies [50] that focused on the examination and detection of the RHDV *VP60* gene from 20 liver homogenates from infected rabbits during an earlier virus outbreak in Egypt (2015 to 2016). In this earlier study, four isolates were among the classical RHDV strains, and only one isolate was identified as a variant. The cases were identified in various Egyptian governorates in 2018-2019. Our findings revealed a high distribution and significant circulation of both the classical and variant virus strains within Egyptian rabbit populations.

DNA sequence analysis resulted in the classification into two genotypes, specifically classic RHDV and variant RHDV2. To the best of our knowledge, these variant RHDV2 strains have not been detected previously in Egypt. As such, this presents a new challenge and threat to the rabbit industry in Egypt. The classical strain was circulating primarily in localities within upper Egypt. However, the new European RHDV2 variant was identified within the governorates of lower Egypt. This result is somewhat to be expected as the lower Egyptian governorates are much closer to some of the European countries such as France, where the variant strain was first recorded. Some authors claim that RHDV2 has become the dominant strain circulating in Europe, and it may have already replaced older RHDV strains and accounts for most of the reported cases in wild and domestic rabbits [12]. The disease associated with RHDV2 variant includes similar manifestations as those seen in response to an infection with one of the classical strains, although mortality rates are variable [13].

The initial characterization of RHDV genetic diversity was initiated by sequencing and comparing partial *VP60* sequences with related isolates from European countries. One research group [16] reported that the classic virus strains typically cause disease in rabbits older than 2 months of age. The results of our study revealed that both genotypes can infect rabbits of different ages. Another previous report [44] noted that clinical cases of acute RHDV from different Egyptian governorates demonstrated similar virulence to cases of previously reported natural RHDV outbreaks. Most of our cases were detected in the summer season, most notably in the month of July, as shown in Figure-3. An earlier report [45] mentioned that RHD had been reported during the spring of 1991 in Sharkia Province, with 90% losses, and also during the winter and spring of 1993 in upper Egypt (Minia and Sohag governorates), with mortality rates of 26.7%-100% in 14-16-old rabbits. Subsequent outbreaks have occurred in other governorates, including Kaliobia [51] and Assuit [52].

## Conclusion

In this study, we reported the detection of two distinct genotypes of RHDV among rabbits of different ages and both genders. We are quite concerned to confirm the emergence and near-dominance of the RHDV2 variant in rabbit populations throughout Egypt. These findings underscore the urgent need to launch a new vaccine that is capable of protection against both circulating genotypes.

## Authors’ Contributions

AME designed this study and applied the molecular analysis. AGS performed molecular biology tests and HA test. Both authors collected samples, drafted, revised the manuscript, analyzed the data, and approved the final manuscript. Both authors read and approved the final manuscript.
